# The Me-File: An Event-Coding Approach to Self-Representation

**DOI:** 10.3389/fpsyg.2021.698778

**Published:** 2021-07-30

**Authors:** Bernhard Hommel

**Affiliations:** ^1^Cognitive Psychology Unit, Institute for Psychological Research & Leiden Institute for Brain and Cognition, Leiden University, Leiden, Netherlands; ^2^Cognitive Neurophysiology, Department of Child and Adolescent Psychiatry, Faculty of Medicine, TU Dresden, Dresden, Germany; ^3^Department of Psychology, Shandong Normal University, Jinan, China

**Keywords:** self representation, agency, body ownership, Theory of Event Coding (TEC), minimal self

## Abstract

Numerous authors have taken it for granted that people represent themselves or even have something like “a self”, but the underlying mechanisms remain a mystery. How do people represent themselves? Here I propose that they do so not any differently from how they represent other individuals, events, and objects: by binding codes representing the sensory consequences of being oneself into a Me-File, that is, into an event file integrating all the codes resulting from the behaving me. This amounts to a Humean bundle-self theory of selfhood, and I will explain how recent extensions of the Theory of Event Coding, a general theory of human perception and action control, provide all the necessary ingredients for specifying the mechanisms underlying such a theory. The Me-File concept is likely to provide a useful mechanistic basis for more specific and more theoretically productive experimentation, as well as for the construction of artificial agents with human-like selves.

## Introduction

Like many other concepts used in academic psychology, the concept of the “self” is rather uncritically taken to refer to something residing in the human mind or brain or both that creates some degree of unity of either the phenomenal experience that we have with or about us or the stories that we are telling about us. Nowhere does one find the concept to be questioned or justified, apparently because both authors and readers consider the existence of a self self-evident (pun partly intended). The reason for this uncritical acceptance is likely to be its philosophical heritage: the only toolbox that philosophers traditionally have available to acquire their data is themselves and their phenomenal experience, so that it does not come as a surprise that the only thing that Descartes was unable to doubt was (the phenomenal experience of) the doubting self. However, less subjective methods did not provide strong support for our intuition that our phenomenal experience plays an important role in our thinking and acting, as it turned out to be too slow and too error-prone to represent a promising causal factor in human perception and action (Nisbett and Wilson, 1977; Wegner, 2002; Hommel, 2013). Moreover, the mere fact that a concept exists in our language cannot be taken as existence proof for a dedicated psychological mechanism responsible for generating the behavior this concept refers to (Danziger, 1997). More specifically, while there is nothing wrong with categorizing all information that receptors provide about the agent carrying them as “belonging to or constituting a self,” the mere fact that this information can be consciously perceived does not yet require any mechanism creating any unity. Along the same lines, the fact that people tend to play the main role in their narratives does not require any dedicated mechanism that makes sure that they do—it may simply be the fact that they happen to be the one they are the most familiar with.

These considerations raise the suspicion that the self-concept carries quite a bit of unnecessary baggage that reflects the natural bias that a limitation of one's empirical toolbox to self-experience brings with it, rather than straightforward functional considerations calling for a dedicated self-mechanism. They also raise the suspicion that many theorists are not yet decided whether they consider the self in its various disguises an explanandum that their theory aims to explain or an explanans that provides this explanation. In fact, many theories try to explain the self as explanandum by referring to some not further explained internal self-system that has no other purpose than generating the explanandum—a clear case of pseudo-explanation (Hommel, 2020). In the following, my aim will be to drop this baggage and develop a purely functional theoretical approach to what we call the self. That is, my aim will be to explain the behavior that theorists consider reflections of a self without referring to a dedicated system producing that behavior. In fact, I will try to do without inventing any new mechanisms to account for such behavior and restrict myself to the Theory of Event Coding (TEC; Hommel et al., 2001; Hommel, 2019a) as my theoretical toolbox.

TEC was conceived as a generic theory of the representations and processes underlying human perception and action. It assumes that perceived and produced events (i.e., action plans) are represented by bindings of codes representing the features of these events, so-called event files (Hommel, 2004). First versions addressed perception and action in very simple tasks involving stimuli with very few features, like red circles and green rectangles, and not overly complex actions, like pressing left and right keys. However, more recent versions addressed more complex tasks and situations (Hommel, 2019a) and questions of self- and other-representation (Hommel, 2018) by means of the same mechanistic principles. Indeed, the representational assumptions of TEC are fully consistent with theoretical frameworks targeting more social processes, including self-representation (Greenwald et al., 2002), which is why I consider the mechanistic toolbox of TEC fully sufficient for understanding self-representation, despite the theory's non-social origin.

## Online and Offline Self

Psychological approaches to the self commonly accept the philosophical distinction between minimal and narrative self. And indeed, it makes intuitive sense to distinguish between Hume's 1739 idea of a personal self consisting of nothing but the perceptual information that an agent has available about herself, so that she in some sense “ceases to exist” when falling asleep, and the idea of an agent who actively sculpts the image of herself by telling self-relevant stories (Gergen and Gergen, 1997; Gallagher, 2000). However, this distinction is heavily confounded with various other factors: the timeframe (second by second versus minutes or years), the medium (perception versus communication), the audience (oneself versus oneself vis-à-vis others), and the reliance on earlier experience, so that it remains unclear whether the distinction between minimal and narrative self actually refers to different concepts, different mechanisms, different kinds of experience, or something else. From a purely functional viewpoint, it seems more reasonable, so I suggest, to distinguish between online and offline self.

### Online Self

The *online self* refers to the here and now, to the flow of information from receptors to more integrative processing levels that inform action control, and vice versa. According to TEC, a person would represent herself just like any other event: by a binding of codes representing the features making up the event, oneself in this particular case. This comprises of all perceivable features regarding oneself in principle, features referring to how one looks, sounds, and smells, but also how one moves and feels—which reflects the ideomotor heritage of TEC, according to which actions and emotions are also grounded in self-perception. Which features belong to this “personal” event may not always be obvious. For instance, infants need quite a while before they develop a good understanding of which objects and events do or do not belong to themselves, and active exploration of their own body and their immediate surrounding plays an important role in this development (for a review, see Verschoor and Hommel, 2017). Even adults can be surprisingly flexible in their self-perception, as indicated by the notorious rubber-hand illusion (Botvinick and Cohen, 1998): when participants are confronted with a rubber hand lying in front of them, simultaneously stroking the rubber hand and the participant's real hand results in the illusion that the rubber hand becomes part of the participant's own body.

These observations suggest that people are not born with a fixed representation of themselves but continuously re-create their self-representation based on the currently available perceptual information. To determine whether perceived features are actually related to themselves or to their physical or social environment, people seem to use the same cues that are known from object perception. For instance, people are more likely to perceive rubber or virtual hands as part of their own body if these artificially effectors are spatially close to their body, if they can be seen as a continuation of their own effectors, and if artificial and real effectors move in synchrony (e.g., Ma and Hommel, 2015). In object and non-social event perception, these kinds of cues are known as the Gestalt laws of spatial and temporal proximity, good Gestalt/continuation, and common fate (Todorovic, 2008), which supports the idea that representing oneself follows the same principles as representing other events. Another well-known principle governing self-perception is the relationship between intended and actual action effects (Hommel, 2015): the event with the closest relationship (i.e., the one that keeps generating action effects that I intend) is probably me (Verschoor and Hommel, 2017). This relationship is an important ingredient of any control system, ranging from central heating to human intentional action (Frith et al., 2000), and presumably the crucial information for judging personal agency (Blakemore et al., 2002).

**Figure 1:**
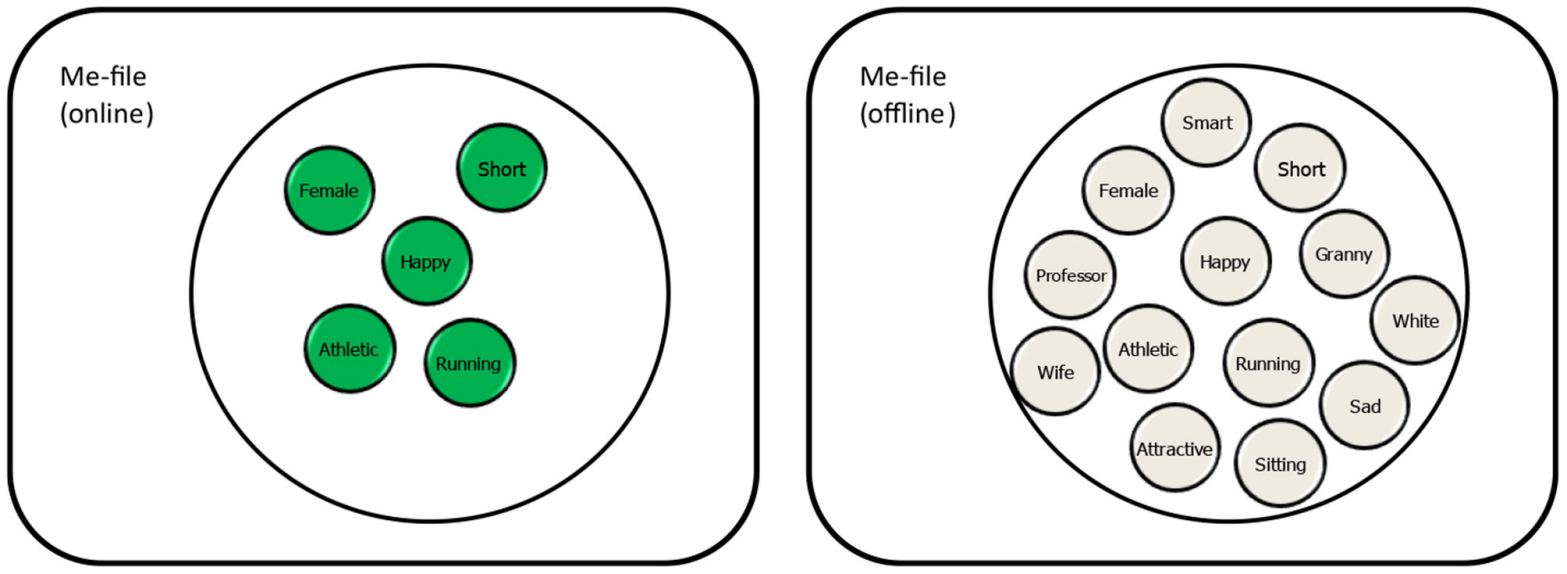
Online and offline Me-files including currently activated (in green, see left panel) and currently inactive (in gray, see right panel) feature codes characterizing the agent.

While the online self can be informed by and interact with stored information (the activated bits of the offline self), it is mainly a reflection of the incoming, currently available information that active agents generate themselves. Accordingly, the binding of the codes that represent the features that specify the active agent—the structure that I will call the Me-file—can be considered to represent the self as envisioned by Hume's bundle-theory, that is, as a direct perceptual reflection of how we currently embody ourselves. Note that this reflection does not distinguish between cognitive, motivational, and affective (or any other kind of) information. As elaborated elsewhere (Hommel, 2019b), such labels refer to different functions of representations and mechanisms but do not necessarily indicate that the underlying representations and mechanisms themselves are separable and specific. For instance, Barrett (2017) has argued that perceived emotion and affect are not generated by dedicated affective mechanisms but derived from general mechanisms with basic survival functions, so that it makes little sense to consider the mechanisms as cognitive, motivational, or affective. Along these lines, the online Me-file of a jogging colleague might look like in the left panel of Figure 1, where going for a jog provides her with feedback about her being busy with running, with being athletic, with being short and female, but also with being happy—among many other features that online feedback might inform about.

### Offline Self

If Hume is right in claiming that people in some sense cease to exist when going asleep, and if this scenario is taken to reflect the fact that we more or less switch off online self-perception during the night, it is easy to see that the online self cannot be all that we have. Obviously, people do not start from scratch in perceiving themselves when waking up, which means that we are able to store perceived information about ourselves in a more durable format—the offline self. As suggested by self-perception theorists like Bem (1972) and Laird (2007), people learn about themselves just like they learn about others: by perceiving their behavior and looking for regularities. I thus do not have privileged information about me being a friendly or aggressive person, say, but I may assume being one if I perceive myself to repeatedly compliment other people or punch them in the face, respectively. Repeatedly making such observations and representing them in my online self is likely to leave traces behind, traces that survive the switching off of my online self during sleep and that provide me with a warm-start the next morning. Accordingly, Greenwald et al. (2002) have suggested that people keep networks of feature codes that refer to one's perceived personal characteristics, like being athletic, intelligent, a professor, grandmother, female, and short, as indicated in the right panel of Figure 1. In contrast to the online self, which is restricted to those feature codes that are currently activated (for reasons discussed in the next section), the offline self refers to the total of all available feature codes that have been involved in self-representation to a degree that they have been bound into a network that represent something like the potential self. In other words, the offline self refers to the knowledge that a person has acquired about herself, about the features that she knows to have in principle.

### Current Self

It is important to emphasize that the terms online self and the offline self do not imply different systems but refer to different levels of activation of feature codes. Cowan (1995) has suggested that short-term memory might be considered the activated part of long-term memory. Hence, whereas long-term memory contains all codes that a person has acquired over the years, only some of these codes are active at any time, irrespective of whether they have been exogenously or endogenously activated, and the total of the currently active codes constitute short-term memory. The same applies to self-representation. The offline self is the total of all feature codes that have become part of the network of codes that the person has learned to represent features of her and that she has used to represent herself in the past. According to TEC, being exposed to a situation, being engaged in a task, and being busy with particular themes increases the *intentional weighting* (Hommel et al., 2001; Memelink and Hommel, 2013) of feature dimensions that the agent considers relevant (based on past experience and current expectations) for making the right choices under these situational circumstances. This means that feature values that are coded on these dimensions are activated more strongly and have a higher impact to impact decision-making and action-selection. If, thus, a participant is asked to press a left versus right key in response to red and green stimuli, respectively, the intentional weighting for color and location will be high, given that these dimensions define the task-relevant aspects of stimuli and responses. Indeed, even preparing for simple tasks like pointing, grasping, and tapping is sufficient to sensitize the agent for attending to and prioritizing stimuli falling onto dimensions that are important for these actions, like location, shape, and rhythm (Schubotz and von Cramon, 2003; Fagioli et al., 2007).

With respect to self-representation, this means that the way we currently represent ourselves is selective and strongly affected by our current concerns and interests, the tasks we carry out, and the situational implications they have. Our *current self* would thus be a mixture of codes that represent currently perceived features of ourselves (the online self), in particular of features related to dimensions that we currently consider relevant, and those feature codes of our offline self that are active for other reasons, perhaps because they are relevant for another task we pursue or intend to pursue in the near future, or because of our current concerns (Klinger and Cox, 2011)—thoughts we are busy with, or because of other needs, like hunger or a need for affiliation (McClelland, 1988; Hommel, 2021). This implies that we are not always the same and do not perceive ourselves as the same under all circumstances. Entering particular social bubbles, like when visiting or family or meeting old friends, is likely to implement different sets of intentional weighting, which in turn will emphasize particular features in our self-perception and deemphasize others. With respect to our example described above, participating in a running competition would increase the intentional weighting for features related to being sporty and fast, so that self-perception would focus on information that is likely to activate the feature codes for being athletic and running, but probably not feature codes for being a professor or a wife.

## Theoretical Implications

In essence, my claim is that people represent themselves like any other event, so that no special theoretical claims need to be made, no novel mechanisms need to be introduced, and no additional assumptions need to be defended, to account for our ability to represent ourselves. And yet, my minimalist account has interesting theoretical implications that can account for numerous empirical observations that have either not been sufficiently well explained so far or that have been explained with specialized, and thus not overly parsimonious theoretical frameworks. In the following, I will briefly touch some of these implications and phenomena they relate to.

### Ownership

My account does not assume any dedicated mechanism responsible for perceiving body ownership, as when being confronted with a body extension, be it a tool or an artificial hand. Instead, it assumes that people judge the degree to which an artificial hand belongs to their body in exactly the same way as they judge the relationship between a dog and its tail: if the tail is close to the dog, if it wiggles only when the dog moves as well, and if it tends to appear and disappear together with the dog, people will perceive the tail as part of the dog. The same applies to a rubber or virtual hand: if it is close to me, if it moves when I move, and if it accompanies me wherever I go, I'm likely to consider it as part of me and my body. Obviously, the informational basis for judging the relationship between oneself and a candidate body part is different from judging the relationship between someone else and a candidate body part: visual information tends to be more comprehensive when observing other agents, whereas interoceptive (kinesthetic, proprioceptive) and tactile information will commonly be available only when perceiving oneself. This may mean that the outcomes of such judgments rely on different kinds of information and may be difficult to compare. Nevertheless, this does not imply any difference in the way the available information is integrated and analyzed, which means that the basic mechanisms and their principles do not differ. It is certainly true that this account does not yet address all theoretical questions. Most importantly, why is it these Gestalt criteria (spatial/temporal proximity, good Gestalt/continuity, and common fate) that people tend to use when judging relationships between events? Are these simply the most reliable indicators or are there cultural or educational factors involved? Tackling such questions is an important challenge for future research, but it is not a question that would be specific for self-representation.

### Agency

Judgments of body ownership and agency tend to be dissociable in the highly artificial rubber-hand scenarios but are strongly correlated in studies with more natural relationships between real body movements and movements of artificial extensions (Ma et al., 2019). This suggests that the informational basis for judging agency and judging body ownership overlaps to a substantial degree. However, there is substantial evidence for a special role of the relationship between personal intentions and related expectations of action outcomes on the one hand and the actual outcomes on the other for judging agency (Hommel, 2015). There is theoretical consensus that information about this relationship can be directly derived from mechanisms underlying action control. Voluntary action is assumed to be selected based on expected action outcomes, which is almost true by definition: given that voluntary action is defined as aiming at particular outcomes, representations of outcomes must play some role in selecting the movements that eventually achieve these outcomes (Hommel, 2009). Moreover, adaptive action control requires insight into the degree to which a particular action has or has not generated the intended action effects, and this insight is commonly derived from comparing expected outcomes with actual outcomes (Frith et al., 2000). It is the result of this comparison that is assumed to contribute to judgments of agency (Blakemore et al., 2002; Chambon and Haggard, 2013; Hommel, 2015), which again means that accounting for agency does not need any dedicated mechanism beyond what has to be assumed for voluntary action control anyway.

### Sticky Intentions

Various authors have pointed out that committing oneself to a goal or intention makes it particularly sticky (Hollenbeck and Klein, 1987). Lewin (1936) suggested that committing oneself to a goal creates a kind of tension in one's cognitive system that seeks for relaxation very much like a biological drive seeks for reduction. Along the same lines, Klinger (2013) suggests that self-commitment turns mere motivation into goal-striving which, among other things, keeps the respective goal active until the intended outcome has been achieved. Commitment to the goal was also considered crucial to engage in actual goal-striving by Locke and colleagues (e.g., Locke et al., 1988) or Gollwitzer and Oettingen (2011), and there is indeed massive evidence suggesting that self-reported commitment to the goal is the central predictor of successful performance, especially in difficult tasks (Hollenbeck and Klein, 1987; Klein et al., 1999). Along the same lines, Goschke and Kuhl (1993) and others demonstrated that concepts that are connected to actual goals are much easier to remember than concepts that are not (intention memory). The authors suggested that this might be due to some special kind of energy that keeps goal-related representations more active than others—but what this special energy (or Lewin's cognitive tension) might consist of remains a mystery.

From a Me-file perspective, the consideration of two well-established mechanistic features of our cognitive system is sufficient to account for sticky intentions. First, preparing for a task allows people to create lasting associations between task-relevant representations. Hence, if, for instance, participants are instructed to carry out action X in response to stimulus A and action Y in response to stimulus B, they seem to create bindings between the representations of A and X and between the representations B and Y even before the very first trial, as witnessed by the observation that, after the instruction has been given, stimuli acquire the power to automatically activate the response they have been assigned to (Meiran et al., 2017). Second, given that every movement of ours provides perceptual feedback about us, our online self is always active, at least as long as we are awake, and so is our current self of which the online self is a part. If so, each feature code that is part of the current self must also be consistently primed to at least some degree, depending on the degree of intentional weighting. Connecting these two considerations suggests that the act that phenomenologically consists in committing to a goal or intention reflects the mechanistic process of merging the representation of this goal/intention with the Me-file (similar to the assumption of Salancik, 1977, that commitment represents a kind of binding between an individual and her actions). As elaborated elsewhere, goals are likely to be represented by criteria that constrain the selection of event files in such a way that goal-consistent actions become more likely to be selected (Hommel and Wiers, 2017; Hommel, 2021). Accordingly, committing to a goal would integrate corresponding selection criteria into the Me-file. As the Me-file tends to be active most of the time, so would the goal criteria, which would explain why not yet achieved goals are sticky—without referring to any metaphorical tension or mysterious energy.

### Self-Symbols

The consideration that associating information with the Me-file could make that information more accessible and increase its impact on selection might also account for a not yet fully understood observation of Sui, Humphreys, and colleagues (e.g., Sui et al., 2012; Sui and Humphreys, 2015). These authors presented participants with arbitrary symbols and asked them to associate these symbols with either themselves, a close relative, or a stranger, before presenting the symbols in simple cognitive tasks. It turned out that the self-related symbol was responded to faster and recall better in various kinds of tasks, suggesting that the simple fact that a symbol was taken to refer to the participant was sufficient to make that symbol enjoy highly prioritized processing. Considering that the instruction to associate a symbol with oneself might consist in integrating that symbol into one's more or less consistently active Me-file would easily account for the reported observations.

### Resting State

The idea of a chronically active Me-file would also fit with the observation that cortical midline regions involved in *resting-state* or *default-mode* activity (i.e., the typical neural activity shown in the absence of a particular task) show strong spatial overlap with regions that are recruited during self-referential processing (D'Argembeau et al., 2005; Qin and Northoff, 2011). The typical instruction in resting-state studies asks participants to engage in no particular task or thought. To the degree that participants follow this instruction, all that remains will be sensory feedback about themselves, which in turn will activate codes that are contained in the Me-file and contribute to the chronically high level of activation of that file. If so, it is easy to understand why this activates areas that are also active during intentional self-referential processing.

### Social Discrimination

Recent political discussions often focus on aspects of social discrimination, be they related to the proper representation or treatment of people with a particular gender, skin color, political or religious orientation, or sexual preference. There are basically two ideas of how discrimination related to any of these features might be overcome: by reducing/eliminating possible or actual attention to the underlying feature dimension (e.g., as implied by the so-called color-blindness theory: Ansell, 2013) or by increasing attention to this dimension (e.g., as claimed by the Woke movement: en.wikipedia.org/wiki/Woke). It might be interesting to mention that my approach suggests concrete hypotheses regarding the processes that these two strategies would evoke and which consequences they would have. Having the goal of attending to skin color would be likely to create a strong association between the codes representing that feature and one's Me-file. This would render skin color an important feature to represent oneself and others, and be likely to make skin color a feature dimension that overshadows other possible dimensions, like those coding for gender, achievement, sociality, and more. Given that discrimination can be positive or negative, depending on one's experience and values, this does not allow predicting the exact consequences. But my approach would predict that Woke principles should increase and stabilize both the absolute and the relative (as compared to other feature dimensions) importance of the targeted feature dimension in perception (of oneself and others), decision-making, and action—which provides a continuous basis for discriminative behavior.

### Individual Differences

The Me-file approach to self-representation provides a novel perspective on inter- and intra-individual differences in self-perception and the impact of self-perception on behavior (or vice versa). As discussed in the previous section, different physical and social contexts are likely to moderate the intentional weighting of both perceptual dimensions and particular context-specific themes. For instance, going to the gym or participating in a sports event in a sense “reduces” the self-perceiving individual to her physical, performance-relevant attributes and abilities, downplaying other aspects, like gender, race, wealth, and academic background, whereas visiting a library will highlight very different attributes and abilities. Spending time with one's peer groups will increase the weight of other perceptual dimensions and themes than spending time with one's parents, which in turn is not unlikely to change one's behavior and the way one perceives oneself. One of the many interesting aspects of these considerations refers to retirement. As discussed by Hommel and Kibele (2016), an important aspect of cognitive aging (i.e., the decline of cognitive abilities with increasing age) is likely to do with what might be called the embodiment of (non-)agency: Retirement is commonly accompanied by a sudden and rather extensive reduction of one's action repertoire and of the opportunities to experience oneself as being an agent that makes active use of this repertoire. The Me-file approach suggests that this must lead to a drastic reduction of the complexity of self-representation, as the individual no longer perceives herself as an active agent in the physical and social world in quite a number of situations—the kind and number of which depends on the particular job one retires from. Hence, not only is the retired individual prevented from actively exercising the cognitive skills the previous job required, but she is also unlearning to perceive herself as someone who does these things: a kind of acquired non-agency. If so, forced retirement might be considered a societal act that undermines personal motivation and self-respect. Other implications refer to upbringing and education. If, as the Me-file suggests, action is such an important ingredient of self-representation, explorative, active learning would not only be mandated for possible educational reasons but also for the building of active self's, that is, for identities that include the agentive aspect of individuals.

## Conclusion

My aim was to present a mechanistically transparent basis for theorizing about the human self. I have used TEC as my theoretical toolbox and argued that no dedicated special assumptions or principles need to be added to account for self-representation. More specifically, I suggest that representing oneself follows the exact same principles as representing others or representing things, even though the type and the amount of information that is available for the resulting representations is likely to differ—for obvious and theoretically not overly relevant reasons, like the fact that some sensory channels provide more information about oneself than about others. I have also suggested that what philosophical approaches have considered the key ingredients of the human self—body ownership and agency—do not require any special theorizing or any dedicated system or mechanism. In fact, reports about body ownership and agency are likely to be based on the same principles that underlie the judgment of relatedness and causality regarding non-personal events, like the motions of billiard balls and, in the case of agency, on comparisons between intended and actual action effects, as available from action-control processes. Hence, what we call the self may not be special at all, and not require any special theorizing. Given that humans are both subjects and objects of research on the self, this may be intellectually disappointing, especially when viewing the issue from the object perspective. However, it does allow us to create mechanistically transparent models that do not require any special modules or systems to account for the selfness aspect of representing ourselves. In particular, the approach allows implementing various aspects of human-like selfhood into various kinds of artificial agents, and even constructing agents that spontaneously acquire their self through sensorimotor experience with their own embodiment.

## Data Availability Statement

The original contributions presented in the study are included in the article/supplementary material, further inquiries can be directed to the corresponding author.

## Author Contributions

The author confirms being the sole contributor of this work and has approved it for publication.

## Conflict of Interest

The author declares that the research was conducted in the absence of any commercial or financial relationships that could be construed as a potential conflict of interest.

## Publisher's Note

All claims expressed in this article are solely those of the authors and do not necessarily represent those of their affiliated organizations, or those of the publisher, the editors and the reviewers. Any product that may be evaluated in this article, or claim that may be made by its manufacturer, is not guaranteed or endorsed by the publisher.

## References

[B1] Ansell A. E. (2013). Race and ethnicity: the key concepts. New York: Routledge. 10.4324/9780203448236

[B2] Barrett L. F. (2017). How Emotions are Made: The Secret Life of the Brain. New York: Houghton Mifflin Harcourt.

[B3] Bem D. J. (1972). Self-perception theory. Adv. Exp. Soc. Psychol. 6, 1–62. 10.1016/S0065-2601(08)60024-6

[B4] Blakemore S. J. Wolpert D. M. Frith C. D. (2002). Abnormalities in the awareness of action. Trends Cogn. Sci. 6, 237–242. 10.1016/S1364-6613(02)01907-1 12039604

[B5] Botvinick M. Cohen J. (1998). Rubber hands ‘feel' touch that eyes see. Nature 391, 756–756. 10.1038/35784 9486643

[B6] Chambon V. Haggard P. (2013). “Premotor or ideomotor: how does the experience of action come about?,” in Action Science: Foundations of an Emerging Discipline, eds Prinz W. Beisert M. Herwig A. (Cambridge, MA: MIT Press), 359–380. 10.7551/mitpress/9780262018555.003.0014

[B7] Cowan N. (1995). Attention and Memory: An Integrated Framework. New York, NY: Oxford University Press.

[B8] Danziger K. (1997). Naming the Mind: How Psychology Found Its Language. Thousand Oaks, CA: Sage Publications.

[B9] D'Argembeau A. Collette F. Van der Linden M. Laureys S. DelFiore G. Degueldre C. (2005) Self-referential reflective activity its relationship with rest: a PET study. Neuroimage 25, 616–624. 10.1016/j.neuroimage.2004.11.048. 15784441

[B10] Fagioli S. Hommel B. Schubotz R. I. (2007). Intentional control of attention: action planning primes action-related stimulus dimensions. Psychol. Res. 71, 22–29. 10.1007/s00426-005-0033-3 16317565

[B11] Frith C. D. Blakemore S. J. Wolpert D. M. (2000). Abnormalities in the awareness and control of action. Philos. Trans. R. Soc. Lond. B: Biol. Sci. 355, 1771–1788. 10.1098/rstb.2000.0734 11205340 PMC1692910

[B12] Gallagher S. (2000). Philosophical conceptions of the self: implications for cognitive science. Trends Cogn. Sci. 4, 14–21. 10.1016/S1364-6613(99)01417-5 10637618

[B13] Gergen K. J. Gergen M. M. (1997). “Narratives of the Self,” in Suny Series in the Philosophy of the Social Sciences. Memory, Identity, Community: The Idea of Narrative in the Human Sciences (New York, NY: State University of New York Press), 161–184.

[B14] Gollwitzer P. M. Oettingen G. (2011). “Planning promotes goal striving,” in Handbook of Self-Regulation: Research, Theory, and Applications, 2nd ed, eds Vohs K. D. Baumeister R. F. (New York: Guilford), 162–185.

[B15] Goschke T. Kuhl J. (1993). Representation of intentions: persisting activation in memory. J. Exp. Psychol. Learn. Memory Cogn. 19, 1211–1226. 10.1037/0278-7393.19.5.1211

[B16] Greenwald A. G. Banaji M. R. Rudman L. A. Farnham S. D. Nosek B. A. Mellott D. S. (2002). A unified theory of implicit attitudes, stereotypes, self-esteem, and self-concept. Psychol. Rev. 109, 3–25. 10.1037/0033-295X.109.1.3 11863040

[B17] Hollenbeck J. R. Klein H. J. (1987). Goal commitment and the goal-setting process: problems, prospects, and proposals for future research. J. Appl. Psychol. 72, 212–220. 10.1037/0021-9010.72.2.212

[B18] Hommel B. (2004). Event files: feature binding in and across perception and action. Trends Cogn. Sci. 8, 494–500. 10.1016/j.tics.2004.08.007 15491903

[B19] Hommel B. (2009). Action control according to TEC (theory of event coding). Psychol. Res. 73, 512–526. 10.1007/s00426-009-0234-2 19337749 PMC2694931

[B20] Hommel B. (2013). Dancing in the dark: no role for consciousness in action control. Front. Psychol. 4:380. 10.3389/fpsyg.2013.00380 23805123 PMC3693078

[B21] Hommel B. (2015). “Action control and the sense of agency,” in The Sense of Agency, eds Haggard P. Eitam B. (New York: Oxford University Press), 307–326. 10.1093/acprof:oso/9780190267278.003.0014

[B22] Hommel B. (2018). Representing oneself and others: an event-coding approach. Exp. Psychol. 65, 323–331. 10.1027/1618-3169/a000433 30638165 PMC6716141

[B23] Hommel B. (2019a). Theory of event coding (TEC) V2.0: representing and controlling perception and action. Atten. Percept. Psychophys. 81, 2139–2154. 10.3758/s13414-019-01779-4 31168699 PMC6848055

[B24] Hommel B. (2019b). Affect and control: a conceptual clarification. Int. J. Psychophysiol. 144, 1–6. 10.1016/j.ijpsycho.2019.07.006 31362029

[B25] Hommel B. (2020). Pseudo-mechanistic explanations in psychology and cognitive neuroscience. Top. Cogn. Sci. 12, 1294–1305. 10.1111/tops.12448 31359621 PMC7687254

[B26] Hommel B. (2021). GOALIATH: A Theory of Goal-Directed Behavior (Submitted). 10.1007/s00426-021-01563-w PMC9090680 34324040

[B27] Hommel B. Kibele A. (2016). Down with retirement: implications of embodied cognition for healthy aging. Front. Psychol. 7:1184. 10.3389/fpsyg.2016.01184 27555831 PMC4977281

[B28] Hommel B. Müsseler J. Aschersleben G. Prinz W. (2001). The theory of event coding (TEC): a framework for perception and action planning. Behav. Brain Sci. 24, 849–878. 10.1017/S0140525X01000103 12239891

[B29] Hommel B. Wiers R. W. (2017). Towards a unitary approach to human action control. Trends Cogn. Sci. 21, 940–949. 10.1016/j.tics.2017.09.009 29150000

[B30] Hume D. (1739). A Treatise of Human Nature. Available online at: https://librivox.org/treatise-of-human-nature-vol-1-by-david-hume (accessed on July 10, 2021).

[B31] Klein H. J. Wesson M. J. Hollenbeck J. R. Alge B. J. (1999). Goal commitment and the goal-setting process: conceptual clarification and empirical synthesis. J. Appl. Psychol. 84, 885–896. 10.1037/0021-9010.84.6.885 10639908

[B32] Klinger E. (2013). Goal commitments and the content of thoughts and dreams: basic principles. Front. Psychol. 10:415. 10.3389/fpsyg.2013.00415 23874312 PMC3708449

[B33] Klinger E. Cox W. M. (2011). “Motivation and the goal theory of current concerns,” in Handbook of Motivational Counseling, 2nd Ed, eds Cox W. M. Klinger E. (Chichester: Wiley), 3–47. 10.1002/9780470979952.ch1

[B34] Laird J. D. (2007). Feelings: The Perception of Self. New York, NY: Oxford University Press. 10.1093/acprof:oso/9780195098891.001.0001

[B35] Lewin K. (1936). Principles of Topological Psychology. New York, NY: McGraw-Hill. 10.1037/10019-000

[B36] Locke E. A. Latham G. P. Erez M. (1988). The determinants of goal commitment. Acad. Manag. Rev. 13, 23–39. 10.5465/amr.1988.4306771

[B37] Ma K. Hommel B. (2015). Body-ownership for actively operated non-corporeal objects. Conscious. Cogn. 36, 75–86. 10.1016/j.concog.2015.06.003 26094223

[B38] Ma K. Hommel B. Cheng H. (2019). The roles of consistency and exclusivity in perceiving body ownership and agency. Psychol. Res. 83, 175–184. 10.1007/s00426-018-0978-7 29362888

[B39] McClelland D. C. (1988). Human Motivation. Cambridge: Cambridge University Press. 10.1017/CBO9781139878289

[B40] Meiran N. Liefooghe B. De Houwer J. (2017). Powerful instructions: automaticity without practice. Curr. Dir. Psychol. Sci. 26, 509–514. 10.1177/0963721417711638

[B41] Memelink J. Hommel B. (2013). Intentional weighting: a basic principle in cognitive control. Psychol. Res. 77, 249–259. 10.1007/s00426-012-0435-y 22526717 PMC3627030

[B42] Nisbett R. Wilson T. (1977). Telling more than we can know: verbal reports on mental processes. Psychol. Rev. 84, 231–259. 10.1037/0033-295X.84.3.231

[B43] Qin P. Northoff G. (2011). How is our self related to midline regions and the default-mode network? Neuroimage 57, 1221–1233. 10.1016/j.neuroimage.2011.05.028 21609772

[B44] Salancik G. R. (1977). Commitment is too easy! Organ. Dyn. 6, 62–80. 10.1016/0090-2616(77)90035-3

[B45] Schubotz R. I. von Cramon D. Y. (2003). Functional-anatomical concepts of human premotor cortex: evidence from fMRI and PET studies. Neuroimage 20, S120–S131. 10.1016/j.neuroimage.2003.09.014 14597305

[B46] Sui J. He X. Humphreys G. W. (2012). Perceptual effects of social salience: evidence from self-prioritization effects on perceptual matching. J. Exp. Psychol. Human Percept. Perform. 38, 1105–1117. 10.1037/a0029792 22963229

[B47] Sui J. Humphreys G. W. (2015). More of me! Distinguishing self and reward bias using redundancy gains. Atten. Percept. Psychophys. 77, 2549–2561. 10.3758/s13414-015-0970-x 26265319

[B48] Todorovic D. (2008). Gestalt principles. Scholarpedia 3:5345. 10.4249/scholarpedia.5345

[B49] Verschoor S. A. Hommel B. (2017). Self-by-doing: the role of action for self-acquisition. Soc. Cogn. 35, 127–145 10.1521/soco.2017.35.2.127

[B50] Wegner D. M. (2002). The Illusion of Conscious Will. Cambridge, MA: MIT Press. 10.7551/mitpress/3650.001.0001

